# Clinical predictors of treatment success following Gasserian ganglion conventional radiofrequency in trigeminal neuralgia: secondary cohort analysis

**DOI:** 10.22514/jofph.2026.065

**Published:** 2026-09-12

**Authors:** Mesut Bakır, Kaan Yavuz, Bedri İlcan, Esra Bayburtluoğlu, Şebnem Rumeli

**Affiliations:** ^1^Pain Clinic, Department of Anesthesiology and Reanimation, Faculty of Medicine, Mersin University, 33343 Mersin, Türkiye

**Keywords:** Trigeminal neuralgia, Gasserian ganglion, Conventional radiofrequency, Predictor, Pain relief, Radiofrequency thermocoagulation

## Abstract

**Background**: The study aimed to identify clinical predictors associated with treatment success following Gasserian ganglion conventional radiofrequency (CRF) in patients with trigeminal neuralgia (TN), with particular focus on the predictive value of early clinical response. **Methods**: This retrospective secondary cohort analysis included 37 patients with classical TN who underwent Gasserian ganglion CRF at a tertiary pain clinic. Treatment success was defined as a ≥50% reduction in Numerical Rating Scale (NRS) score at 6 months compared with baseline. Demographic characteristics, baseline pain severity, trigeminal branch involvement, early post-procedural pain response, complications, additional treatment requirements, and patient satisfaction scores were analyzed. Receiver operating characteristic (ROC) analysis was performed to evaluate the predictive performance of early pain reduction for 6-month treatment success. **Results**: Twenty-nine patients (78.4%) achieved treatment success at 6 months. The success group demonstrated significantly higher median age (67 *vs.* 54.5 years, *p* = 0.006) and baseline pain intensity (NRS0: 10 *vs.* 8, *p* = 0.007) compared with the non-success group. Early treatment response at 1 month was strongly associated with 6-month success. Median percentage reduction in NRS score at 1 month was significantly greater in the success group (60% *vs.* 31.25%, *p* = 0.008). Achieving ≥50% pain reduction at 1 month was significantly associated with treatment success at 6 months (*p* = 0.0005). ROC analysis demonstrated good predictive performance for early pain reduction (area under the curve (AUC) = 0.804), with a sensitivity of 82.8% and specificity of 87.5% for the ≥50% cutoff. Patient satisfaction scores were higher among patients with treatment success (*p* < 0.001). **Conclusions**: Early post-procedural pain reduction appears to be a clinically useful marker of sustained mid-term treatment success after Gasserian ganglion CRF in TN. In particular, a ≥50% reduction in NRS score at 1 month may help identify patients likely to maintain meaningful pain relief at 6 months.

## 1. Introduction

Trigeminal neuralgia (TN) is a debilitating neuropathic pain disorder characterized by recurrent, sudden, electric shock-like facial pain involving one or more branches of the trigeminal nerve [1]. The condition substantially impairs daily functioning, psychological well-being, and overall quality of life [1]. Although pharmacological treatment with carbamazepine or oxcarbazepine remains the first-line therapeutic approach, many patients eventually experience insufficient pain control, adverse drug effects, or treatment intolerance, leading to the need for interventional management strategies [2].

Among the minimally invasive interventional procedures, Gasserian ganglion conventional radiofrequency (CRF) is widely used for medically refractory TN because of its ability to provide substantial and sustained pain relief [3]. By creating controlled thermocoagulation of nociceptive fibers within the trigeminal ganglion, CRF can achieve meaningful long-term analgesia with acceptable complication rates [4]. Although Gasserian ganglion CRF provides substantial and sustained pain relief in patients with medically refractory TN, procedure-related adverse effects—including facial hypoesthesia, corneal hypoesthesia, dysesthesia, and masticatory muscle weakness—have been reported in the literature [5]. However, treatment response after CRF is not uniform, and some patients experience insufficient or short-lived benefit despite technically successful procedures [6].

Although Gasserian CRF is widely used in medically refractory TN, treatment response remains heterogeneous, and predictors of sustained clinical benefit are still not clearly defined [7]. Most previous studies have primarily focused on procedural efficacy, recurrence rates, or complication profiles rather than identifying early clinical markers associated with long-term treatment success [8]. In particular, the predictive value of early post-procedural pain response for subsequent mid-term outcomes remains insufficiently characterized in routine clinical practice.

In our previously published retrospective cohort study comparing Gasserian ganglion CRF and peripheral pulsed radiofrequency (PRF) in TN, Gasserian CRF demonstrated superior mid-term pain relief and higher patient satisfaction [9]. However, that study was primarily designed as a comparative effectiveness analysis and did not specifically evaluate patient-level predictors of successful outcome within the CRF cohort.

Therefore, the aim of the present secondary cohort analysis was to identify clinical predictors associated with treatment success following Gasserian ganglion CRF in patients with TN, with particular focus on the predictive value of early clinical response.

## 2. Materials and methods

### 2.1 Study design and participants

This retrospective secondary analysis was conducted at the Pain Clinic, Mersin University Faculty of Medicine. The study is based on a previously published cohort of patients with classical TN who underwent radiofrequency procedures between January 2015 and June 2025. The original study protocol was approved by the Mersin University Clinical Research Ethics Committee (Approval No: 2025/606, Date: 28 May 2025). The present secondary analysis of the same anonymized dataset was subsequently reviewed and determined to be ethically appropriate by the Mersin University Health Sciences Research Ethics Committee (Document No. E-59760191-050.04-3597223).

The present study was specifically designed to evaluate predictors of treatment success within the CRF cohort.

Inclusion criteria were: age ≥18 years; diagnosis of classical TN according to the International Classification of Headache Disorders, 3rd edition (ICHD-3); failure to respond to medical therapy for at least six months; and availability of complete clinical and follow-up data for at least six months.

Exclusion criteria included secondary TN due to structural causes, prior craniofacial or neuroablative procedures, and incomplete clinical or follow-up records.

### 2.2 Procedure

Gasserian ganglion CRF ablation was performed under fluoroscopic guidance in the operating room using 100-mm radiofrequency cannulas with 5-mm active tips. The procedure was carried out under deep sedation. Following confirmation of appropriate needle placement using sensory and motor stimulation, thermal lesioning was applied at 65–75 °C for 60–90 seconds according to standard institutional practice and individual clinical judgment (Fig. 1).

**Fig. 1.** Representative fluoroscopic anteroposterior (A) and lateral (B) views obtained during Gasserian ganglion conventional radiofrequency cannulation through the foramen ovale. In the anteroposterior view, the arrow indicates the foramen ovale and fluoroscopic tunnel view of the radiofrequency cannula. In the lateral view, the arrow indicates the needle tip positioned adjacent to the Gasserian ganglion.

### 2.3 Data collection

Demographic data (age, sex), clinical characteristics (affected side and trigeminal nerve branch involvement), procedural variables, complications, additional treatment requirements, and outcome measures were extracted from patient records.

Pain intensity was assessed using the Numerical Rating Scale (NRS) at baseline and at 1, 3, and 6 months following the procedure.

Patient satisfaction was assessed using a 5-item Likert scale, as defined in the original study, for patients with available records. Each item was rated on a 5-point scale, and total scores were calculated to reflect overall satisfaction.

Only variables available in the original dataset were included in the present analysis.

### 2.4 Outcome measures

The primary outcome was treatment success, defined as a ≥50% reduction in NRS score at 6 months compared with baseline.

Secondary outcomes included:

● The association between early treatment response at 1 month and 6-month treatment success,

● Predictive performance of early pain reduction,

● Patient satisfaction scores, and

● Complication rates.

### 2.5 Statistical analysis

Data were analyzed using SPSS version 22.0 (IBM Corp., Armonk, NY, USA). Continuous variables were assessed for normality and presented as mean ± standard deviation (SD) or median (min–max), as appropriate. Categorical variables were expressed as frequencies and percentages.

Comparisons between patients with and without treatment success were performed using the Student’s *t*-test or Mann-Whitney U test for continuous variables and the Chi-square or Fisher’s exact test for categorical variables.

Receiver operating characteristic (ROC) curve analysis was performed to evaluate the predictive performance of early treatment response for 6-month treatment success. The optimal cutoff value was determined using the Youden index.

A *p*-value < 0.05 was considered statistically significant.

## 3. Results

### 3.1 Baseline characteristics and treatment success

A total of 37 patients who underwent Gasserian ganglion CRF were included in the analysis. Based on the predefined criterion of ≥50% reduction in NRS score at 6 months, 29 patients (78.4%) were classified as having treatment success, while 8 patients (21.6%) were classified as unsuccessful.

Baseline demographic and clinical characteristics were compared between patients with and without treatment success. The success group had a significantly higher median age compared with the non-success group (67 *vs.* 54.5 years, *p* = 0.006). Baseline pain intensity (NRS0) was also significantly higher in the success group (median: 10 *vs.* 8, *p* = 0.007).

There were no statistically significant differences between the groups in terms of sex distribution (*p* = 0.685) or affected side (*p* = 0.109). Trigeminal nerve branch involvement showed a trend toward association with treatment outcome; however, this did not reach statistical significance (*p* = 0.428). Baseline demographic and clinical characteristics according to treatment success are summarized in Table 1.

**Table 1.** Baseline characteristics according to treatment success.

Variable	Success (n = 29)	Non-success (n = 8)	*p*-value
Age (yr), median, mean ± SD	67 (59–73), 65.9 ± 9.5	54.5 (49–60), 52.8 ± 11.9	0.006
Female sex, n (%)	19 (65.5%)	6 (75.0%)	0.685
Right-sided TN, n (%)	15 (51.7%)	7 (87.5%)	0.109
V2–3 involvement, n (%)	18 (62.1%)	2 (25.0%)	0.428

*SD: standard deviation; TN: trigeminal neuralgia.*

### 3.2 Early treatment response and predictive performance

A significant association was observed between early treatment response at 1 month and treatment success at 6 months. Patients in the success group demonstrated significantly lower NRS1 scores compared with the non-success group (median: 4 *vs.* 5.5, *p* = 0.035).

When expressed as percentage reduction from baseline, early response was significantly greater in the success group (median reduction: 60% *vs.* 31.25%, *p* = 0.008). Moreover, achieving ≥50% pain reduction at 1 month was significantly associated with treatment success at 6 months (*p* = 0.0005). Early treatment response variables associated with treatment success are presented in Table 2.

**Table 2.** Early treatment response according to 6-month treatment success.

Variable	Success (n = 29)	Non-success (n = 8)	*p* value
Baseline NRS	10.00 (8.00–10.00) 9.28 ± 0.88	8.00 (8.00–8.25) 8.25 ± 0.46	0.007
1-month NRS	4.0 (2.0–5.0) 3.90 ± 2.43	5.5 (5.0–7.3) 5.75 ± 1.98	0.035
1-month pain reduction (%)	60.0 (50.0–75.0) 57.9 ± 26.5	31.3 (9.4–44.4) 29.9 ± 25.7	0.008

*NRS: Numerical Rating Scale.*

ROC analysis demonstrated that percentage reduction in NRS score at 1 month had good predictive ability for treatment success at 6 months, with an area under the curve (AUC) of 0.804 (95% confidence interval (CI): 0.653–0.955). A ≥50% reduction in NRS score at 1 month yielded the optimal cutoff value, with a sensitivity of 82.8% (95% CI: 64.2%–94.2%) and specificity of 87.5% (95% CI: 47.3%–99.7%) for predicting treatment success. The ROC curve is presented in Fig. 2. Effect sizes for significant group differences ranged from moderate to large, including age (*r* = 0.44), baseline NRS (*r* = 0.46), 1-month NRS (*r* = 0.35), and 1-month pain reduction (*r* = 0.43).

**Fig. 2.** Receiver operating characteristic (ROC) curve for percentage reduction in NRS score at 1 month predicting treatment success at 6 months. AUC: area under the curve; NRS: Numerical Rating Scale.

### 3.3 Patient satisfaction and complications

Median Likert satisfaction scores were significantly higher in the success group compared with the non-success group (25 *vs.* 15, *p* < 0.001; mean ± SD: 24.0 ± 2.1 *vs.* 13.8 ± 5.8; effect size *r* = 0.77). Patient satisfaction was analyzed descriptively and was not included as a predictive variable because it represents a post-treatment outcome measure.

Additional treatment requirements and complication rates were also descriptively evaluated. Minor complications included facial hypoesthesia in one patient (2.7%), corneal hypoesthesia in two patients (5.4%), and transient masseter weakness in three patients (8.1%). No major complications were observed. Due to the limited number of adverse events, comparative statistical analysis was not performed.

## 4. Discussion

This secondary cohort analysis identified several clinically relevant predictors of treatment success after Gasserian ganglion CRF in patients with TN. The main finding was that early clinical response at 1 month was significantly associated with 6-month treatment success. In particular, a ≥50% reduction in NRS at 1 month showed good predictive performance, with an AUC of 0.804, sensitivity of 82.8%, and specificity of 87.5%. These findings suggest that early post-procedural pain reduction may serve as a practical marker for identifying patients likely to maintain mid-term benefit.

The overall success rate in the present cohort was 78.4%, which is consistent with recent real-world studies reporting meaningful pain relief after percutaneous radiofrequency procedures for TN. Yang *et al*. [10] evaluated 392 patients undergoing percutaneous radiofrequency trigeminal rhizotomy and reported significant pain improvement, supporting RF as an effective option in refractory cases.

Higher baseline pain intensity in the success group may reflect greater measurable room for clinical improvement following Gasserian ganglion CRF. Similar observations have been reported in previous radiofrequency studies in TN, in which patients with more severe baseline pain demonstrated substantial post-procedural pain reduction despite variability in long-term outcomes [11]. These findings support the potential clinical relevance of baseline pain severity when interpreting treatment response after RF interventions. However, this observation should be interpreted cautiously given the limited sample size of the present cohort.

Age was also significantly associated with treatment success in our cohort, with older patients demonstrating better outcomes. This may be clinically relevant because Gasserian RF is often selected for elderly or medically fragile patients who may not be optimal candidates for more invasive surgical procedures. Recent studies in elderly TN populations have similarly emphasized percutaneous radiofrequency thermocoagulation as an effective and safe treatment option, including in settings where surgical alternatives may be limited [6, 12, 13].

Trigeminal branch distribution showed a trend toward association with treatment outcome, although it did not reach statistical significance. This finding should be interpreted cautiously due to the small sample size and uneven distribution of affected branches. In a large retrospective analysis of 1070 patients with maxillary trigeminal neuralgia, Zhao *et al*. [7] reported favorable long-term outcomes after Gasserian ganglion radiofrequency thermocoagulation (RFT) and evaluated predictors of long-term pain relief and complications, highlighting the importance of anatomical distribution in RF outcomes.

Recent comparative studies have highlighted differences in clinical outcomes between trigeminal radiofrequency techniques [14]. In our previously published cohort, Gasserian CRF demonstrated superior mid-term pain relief compared with peripheral PRF. The present study extends those findings by focusing specifically on predictors of successful outcome within the CRF cohort.

Patient satisfaction was significantly higher among patients with treatment success. This finding supports the clinical relevance of the predefined NRS-based success criterion. Similar associations between pain reduction and patient-reported satisfaction have been emphasized in previous studies evaluating interventional treatments for TN and chronic pain conditions, supporting the clinical relevance of patient-centered outcome measures in pain research [15, 16]. This observation also supports the construct validity of the predefined treatment success definition used in the present study. However, satisfaction was not included in the predictive model because it represents a post-treatment outcome rather than a baseline or early clinical marker.

The main clinical implication of this study is that 1-month response may be used as an early decision point after Gasserian ganglion CRF. Patients who fail to achieve meaningful early pain reduction may require closer follow-up, medication optimization, or timely consideration of alternative interventions. Conversely, patients achieving ≥50% pain reduction at 1 month are likely to maintain successful outcomes at 6 months. Similar observations have been reported in previous studies evaluating radiofrequency procedures for TN, in which early post-procedural pain relief was associated with sustained clinical benefit during follow-up [17, 18]. This supports the potential value of early response assessment in guiding post-procedural management and patient counseling.

This study has several limitations. First, it was a retrospective secondary analysis with a relatively small sample size, particularly a limited number of unsuccessful outcomes, which reduced statistical power and precluded robust multivariable modeling. Therefore, the identified variables should not be interpreted as independent predictors, and the findings should be considered exploratory and hypothesis-generating rather than definitive. Second, the primary predictor (1-month pain reduction) and the primary outcome (6-month pain reduction) represent repeated assessments of the same clinical construct. Although early clinical response is highly relevant for patient management, this relationship should not be interpreted as establishing causality. Third, the proposed 50% cutoff and its predictive performance were derived and evaluated within the same dataset without internal or external validation, and therefore require confirmation in larger prospective cohorts before being adopted in routine clinical practice. In addition, lesion temperature and duration were individualized within the accepted institutional practice according to clinical judgment, and this procedural variability may have influenced treatment outcomes. Finally, the study was conducted at a single center, follow-up was limited to 6 months, and anatomical subgroup analyses were underpowered, which may limit the generalizability of the findings.

## 5. Conclusions

In conclusion, early post-procedural pain reduction appears to be a clinically useful marker of sustained mid-term treatment success after Gasserian ganglion CRF in TN. In particular, a ≥50% reduction in NRS score at 1 month may help identify patients likely to maintain meaningful pain relief at 6 months. Assessment of early treatment response may therefore provide a practical clinical decision point for post-procedural follow-up, allowing patients with insufficient early improvement to be identified for closer monitoring, optimization of medical therapy, or consideration of alternative treatment strategies. However, given the retrospective design, small sample size, and lack of validation of the proposed cutoff, these findings should be considered exploratory and require confirmation in larger prospective cohorts before routine clinical implementation.
